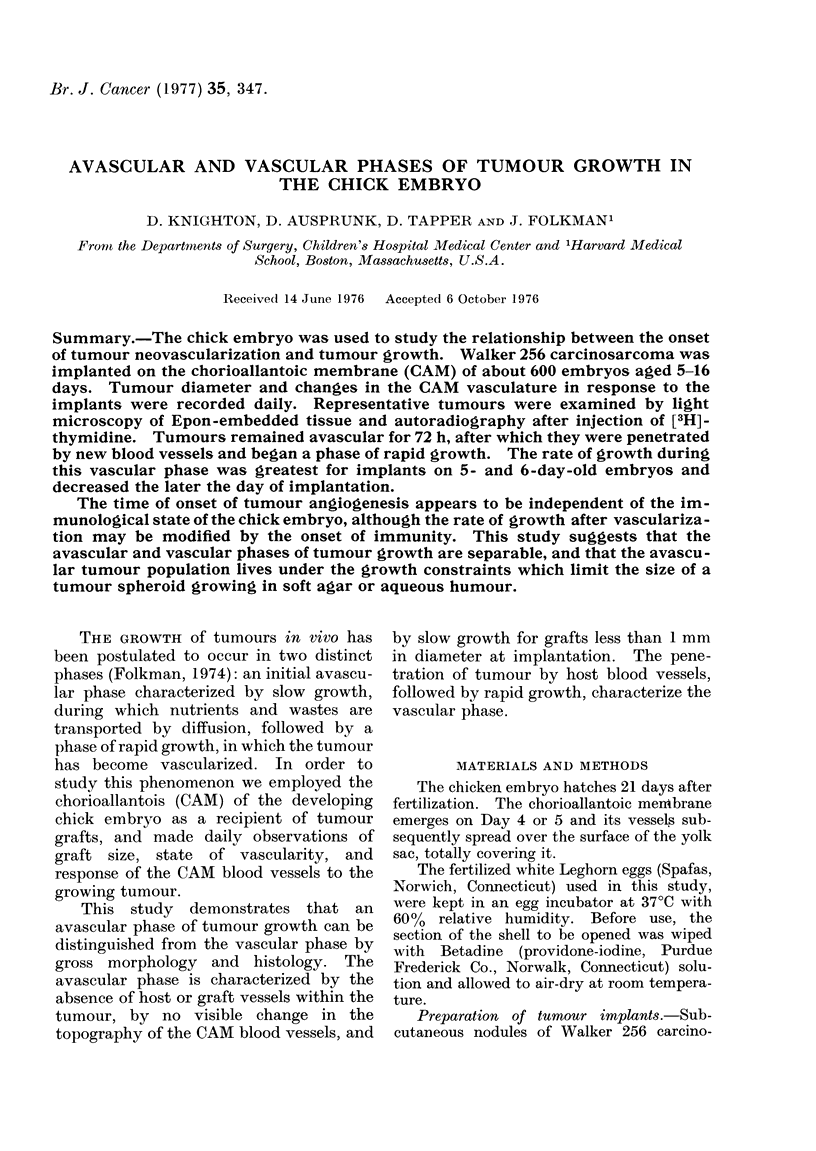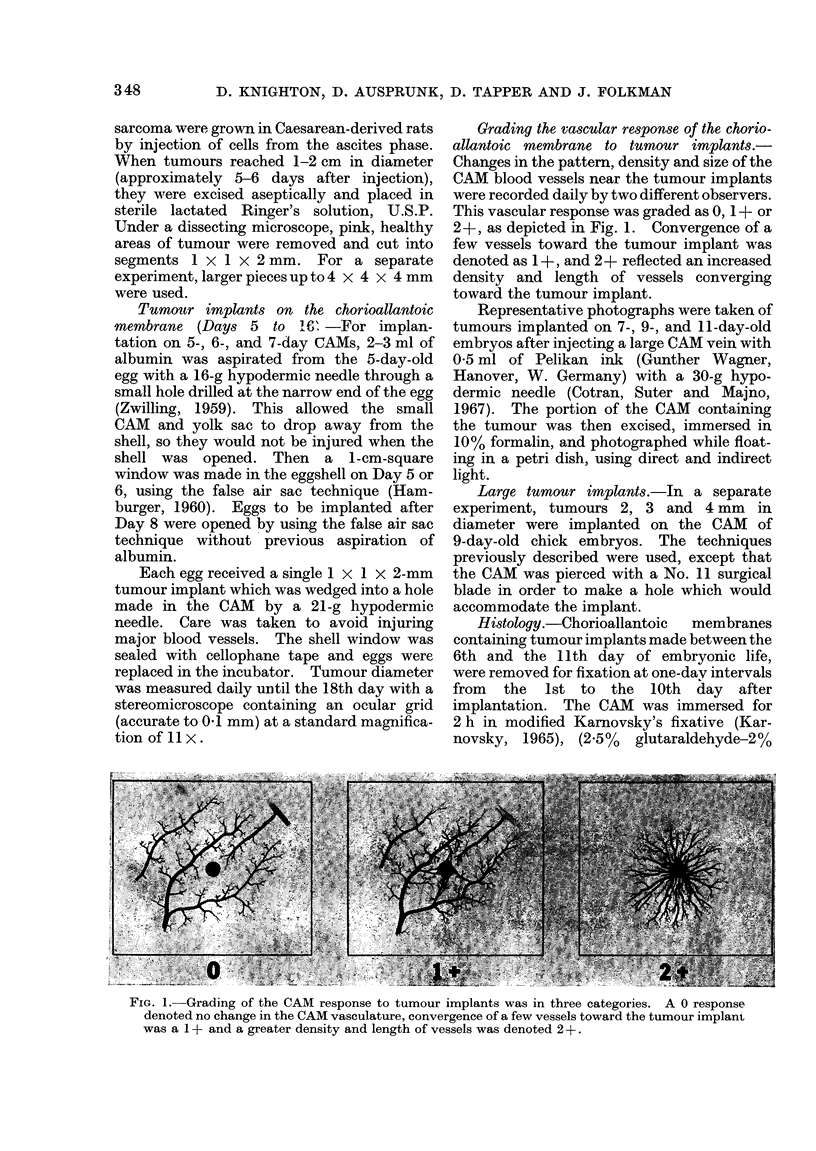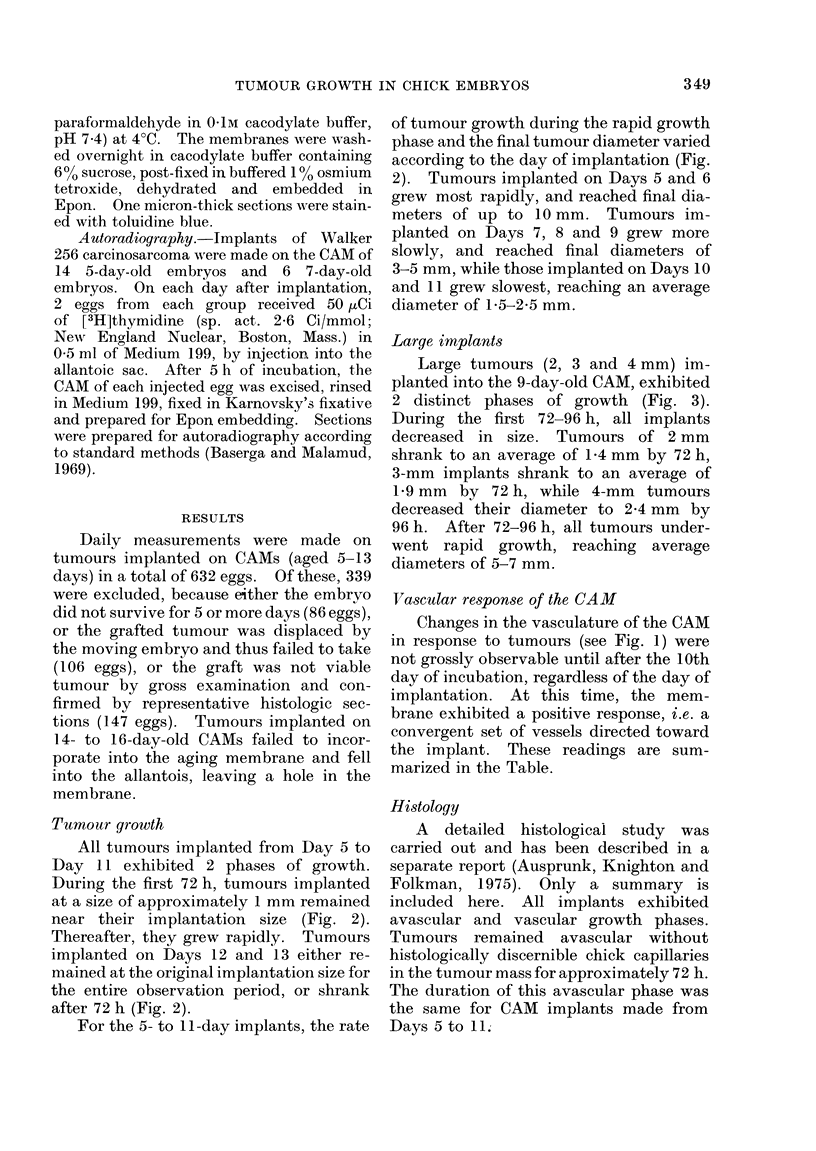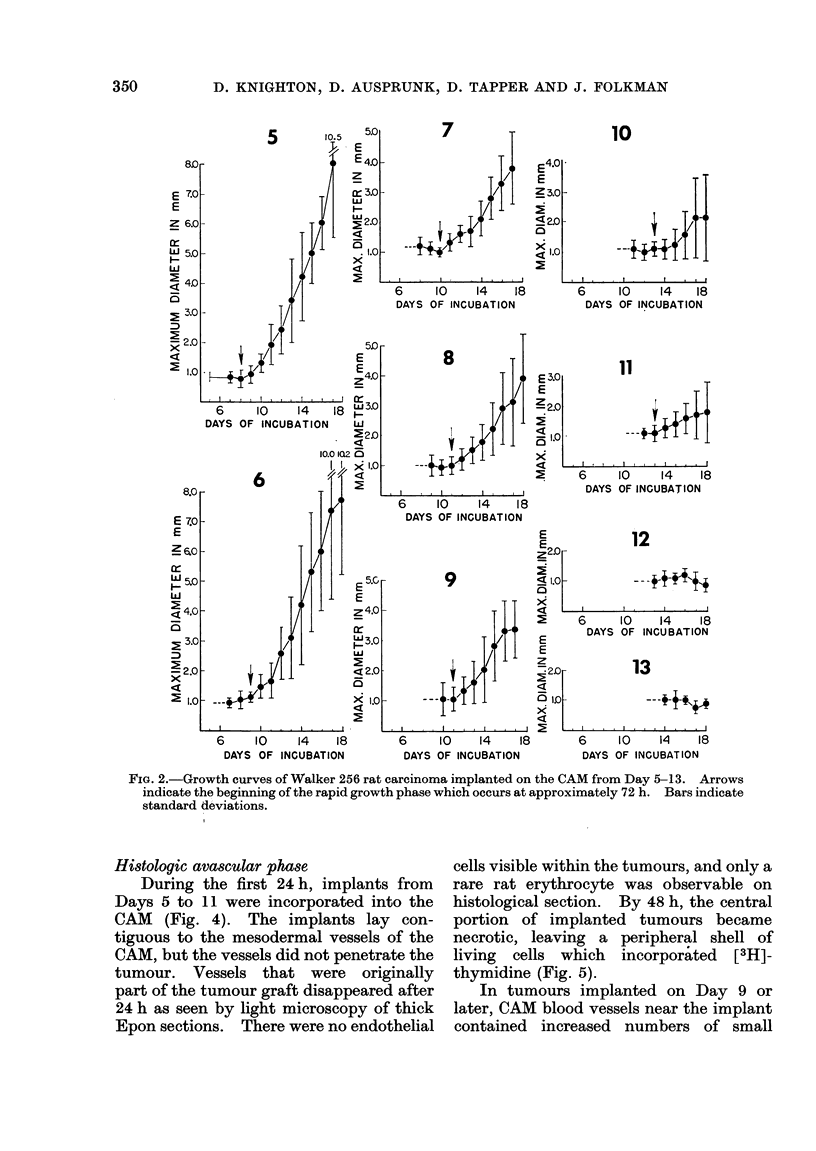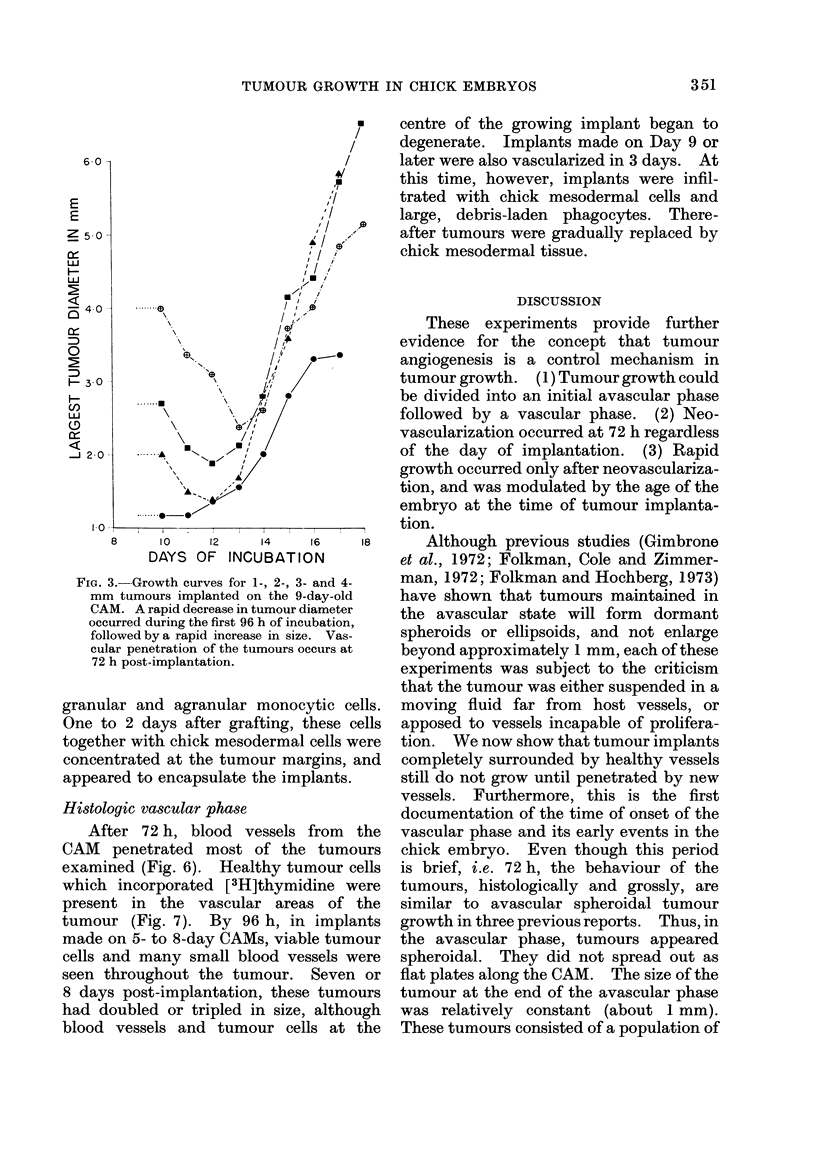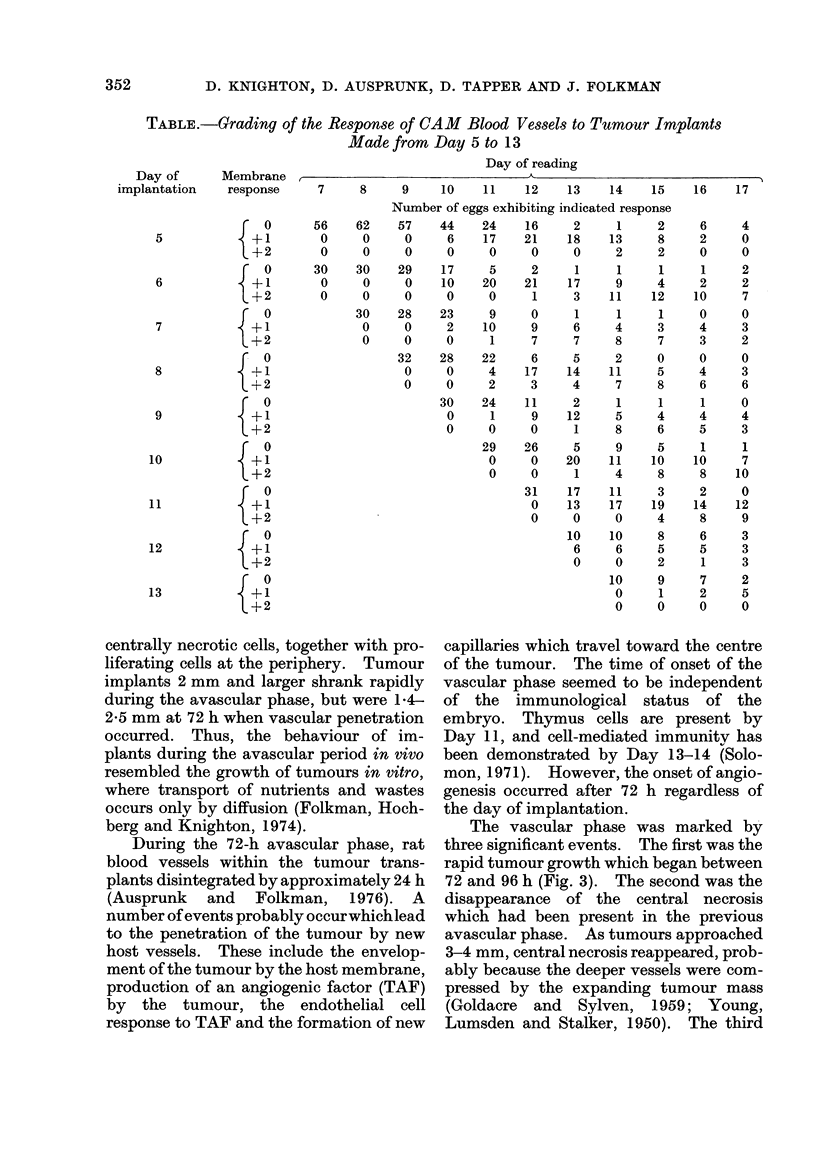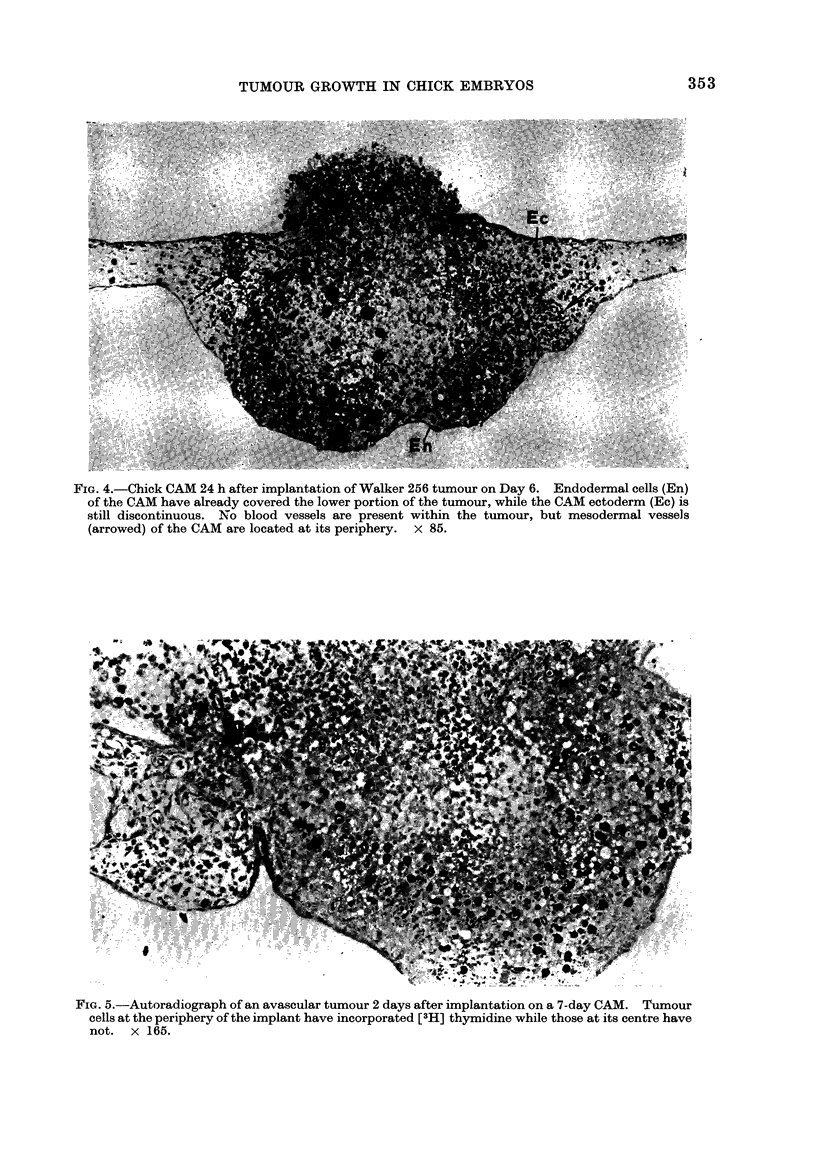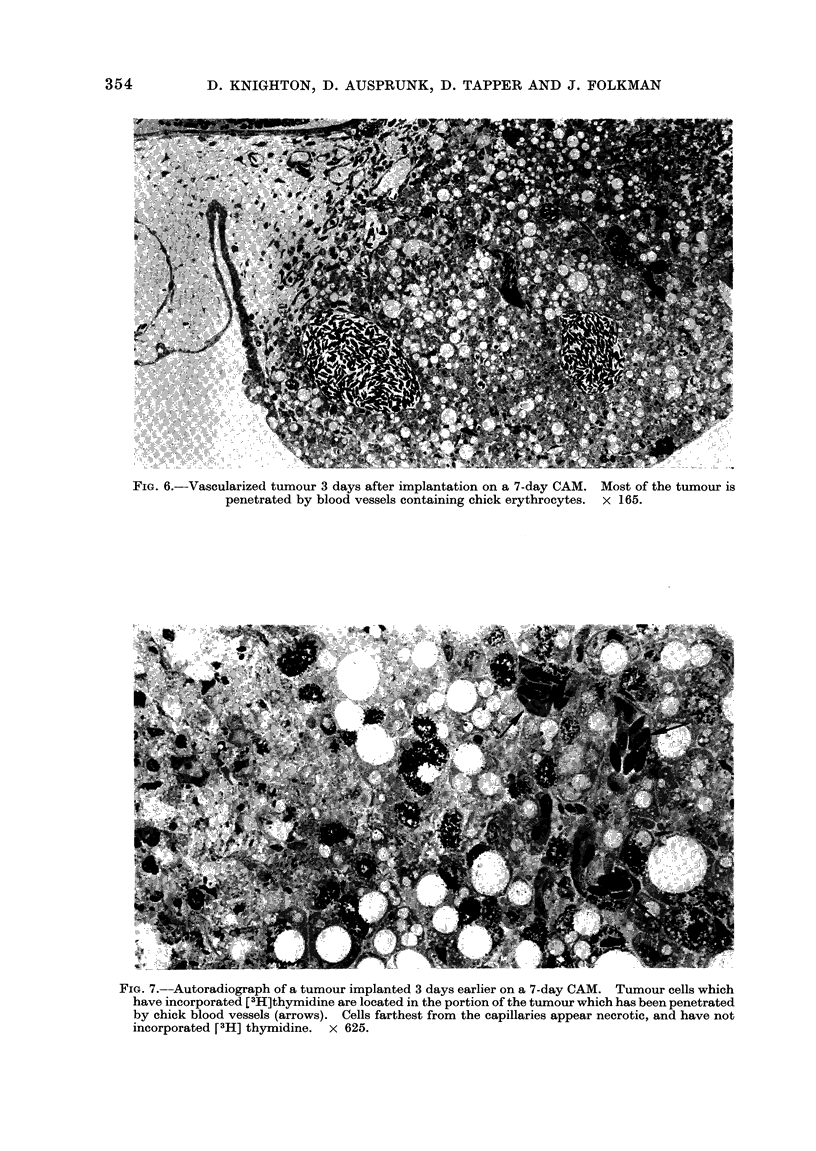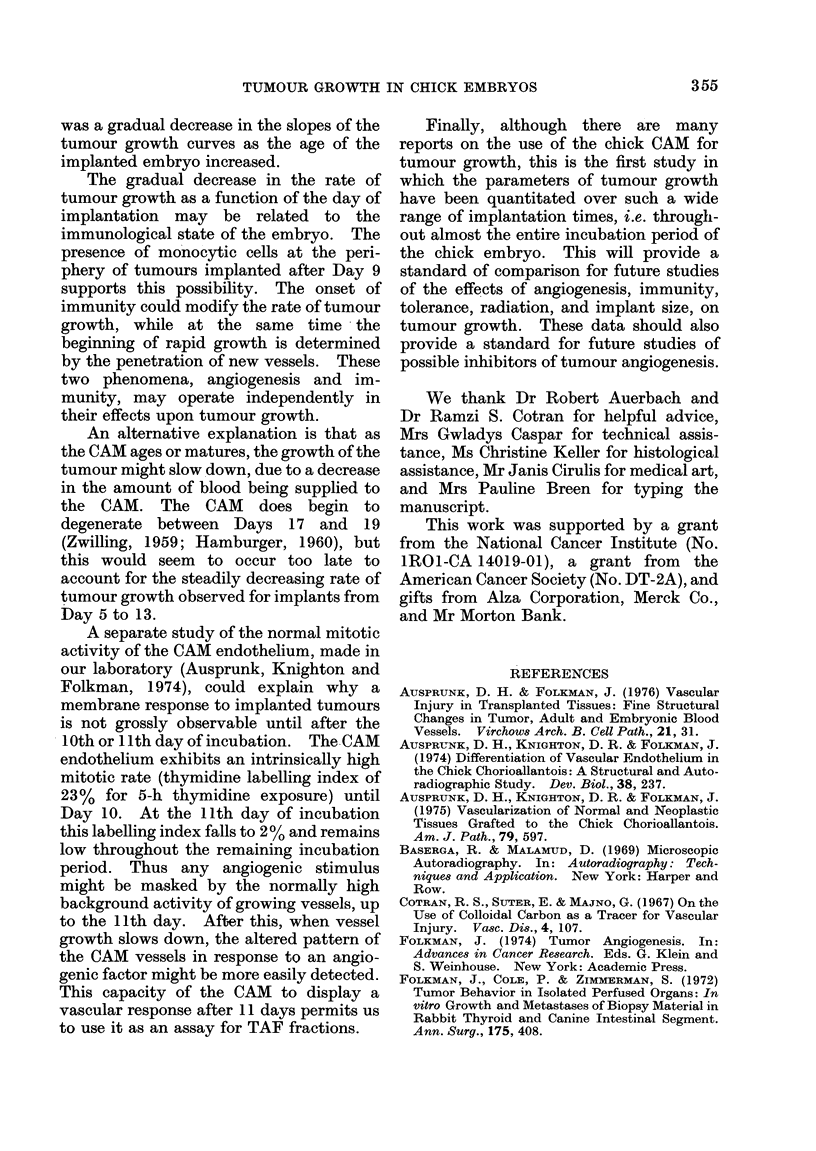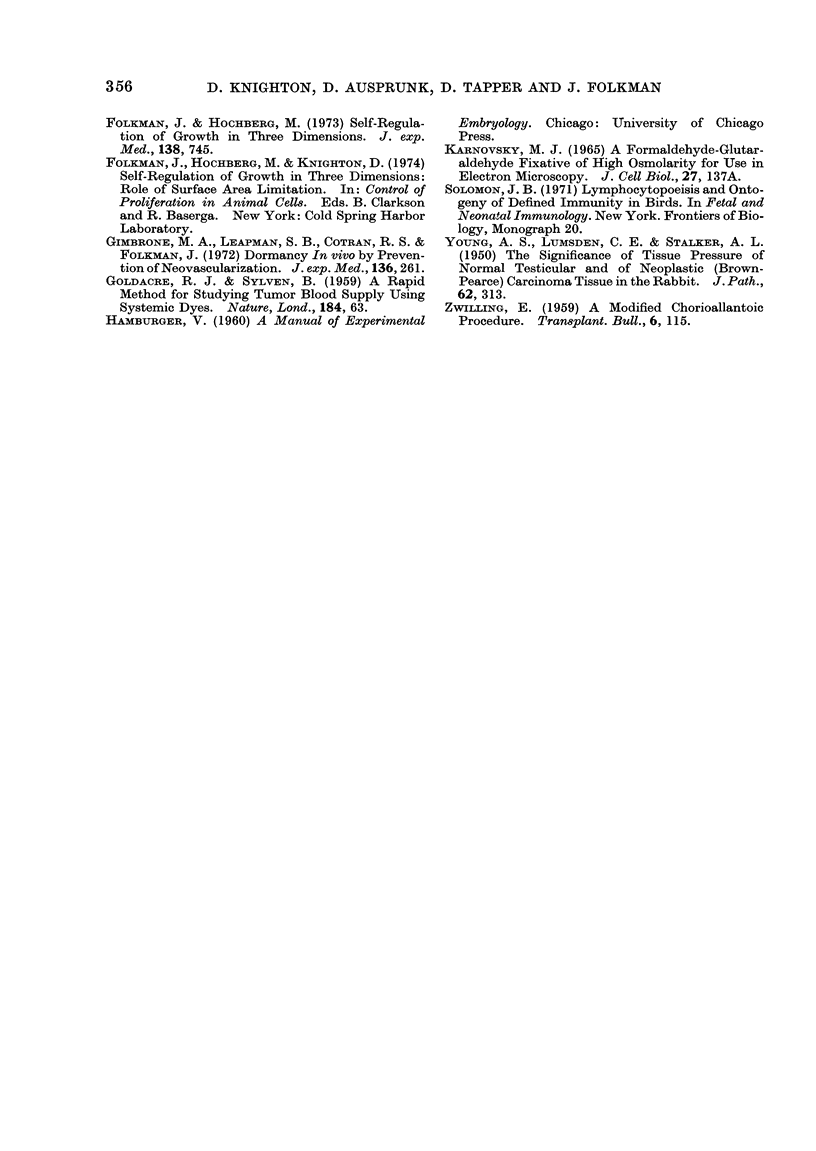# Avascular and vascular phases of tumour growth in the chick embryo.

**DOI:** 10.1038/bjc.1977.49

**Published:** 1977-03

**Authors:** D. Knighton, D. Ausprunk, D. Tapper, J. Folkman

## Abstract

**Images:**


*Br. J. Cancer* (1977) **35**, 347.

### AVASCULAR AND VASCULAR PHASES OF TUMOUR GROWTH IN THE CHICK EMBRYO

D. KNIGHTON, D. AUSPRUNK, D. TAPPER AND J. FOLKMAN[1]

*From the Departments of Surgery, Children's Hospital Medical Center and [1]Harvard Medical School, Boston, Massachusetts, U.S.A.*

Received 14 June 1976 Accepted 6 October 1976

**Summary.—The chick embryo was used to study the relationship between the onset of tumour neovascularization and tumour growth. Walker 256 carcinosarcoma was implanted on the chorioallantoic membrane (CAM) of about 600 embryos aged 5–16 days. Tumour diameter and changes in the CAM vasculature in response to the implants were recorded daily. Representative tumours were examined by light microscopy of Epon-embedded tissue and autoradiography after injection of [$^3$H]-thymidine. Tumours remained avascular for 72 h, after which they were penetrated by new blood vessels and began a phase of rapid growth. The rate of growth during this vascular phase was greatest for implants on 5- and 6-day-old embryos and decreased the later the day of implantation.**

**The time of onset of tumour angiogenesis appears to be independent of the immunological state of the chick embryo, although the rate of growth after vascularization may be modified by the onset of immunity. This study suggests that the avascular and vascular phases of tumour growth are separable, and that the avascular tumour population lives under the growth constraints which limit the size of a tumour spheroid growing in soft agar or aqueous humour.**

THE GROWTH of tumours *in vivo* has been postulated to occur in two distinct phases (Folkman, 1974): an initial avascular phase characterized by slow growth, during which nutrients and wastes are transported by diffusion, followed by a phase of rapid growth, in which the tumour has become vascularized. In order to study this phenomenon we employed the chorioallantois (CAM) of the developing chick embryo as a recipient of tumour grafts, and made daily observations of graft size, state of vascularity, and response of the CAM blood vessels to the growing tumour.

This study demonstrates that an avascular phase of tumour growth can be distinguished from the vascular phase by gross morphology and histology. The avascular phase is characterized by the absence of host or graft vessels within the tumour, by no visible change in the topography of the CAM blood vessels, and by slow growth for grafts less than 1 mm in diameter at implantation. The penetration of tumour by host blood vessels, followed by rapid growth, characterize the vascular phase.

### MATERIALS AND METHODS

The chicken embryo hatches 21 days after fertilization. The chorioallantoic membrane emerges on Day 4 or 5 and its vessels subsequently spread over the surface of the yolk sac, totally covering it.

The fertilized white Leghorn eggs (Spafas, Norwich, Connecticut) used in this study, were kept in an egg incubator at 37°C with 60% relative humidity. Before use, the section of the shell to be opened was wiped with Betadine (providone-iodine, Purdue Frederick Co., Norwalk, Connecticut) solution and allowed to air-dry at room temperature.

*Preparation of tumour implants.*—Subcutaneous nodules of Walker 256 carcinosarcoma were grown in Caesarean-derived rats by injection of cells from the ascites phase. When tumours reached 1–2 cm in diameter (approximately 5–6 days after injection), they were excised aseptically and placed in sterile lactated Ringer's solution, U.S.P. Under a dissecting microscope, pink, healthy areas of tumour were removed and cut into segments 1 × 1 × 2 mm. For a separate experiment, larger pieces up to 4 × 4 × 4 mm were used.

*Tumour implants on the chorioallantoic membrane (Days 5 to 16)*.—For implantation on 5-, 6-, and 7-day CAMs, 2–3 ml of albumin was aspirated from the 5-day-old egg with a 16-g hypodermic needle through a small hole drilled at the narrow end of the egg (Zwilling, 1959). This allowed the small CAM and yolk sac to drop away from the shell, so they would not be injured when the shell was opened. Then a 1-cm-square window was made in the eggshell on Day 5 or 6, using the false air sac technique (Hamburger, 1960). Eggs to be implanted after Day 8 were opened by using the false air sac technique without previous aspiration of albumin.

Each egg received a single 1 × 1 × 2-mm tumour implant which was wedged into a hole made in the CAM by a 21-g hypodermic needle. Care was taken to avoid injuring major blood vessels. The shell window was sealed with cellophane tape and eggs were replaced in the incubator. Tumour diameter was measured daily until the 18th day with a stereomicroscope containing an ocular grid (accurate to 0·1 mm) at a standard magnification of 11×.

*Grading the vascular response of the chorioallantoic membrane to tumour implants*.—Changes in the pattern, density and size of the CAM blood vessels near the tumour implants were recorded daily by two different observers. This vascular response was graded as 0, 1+ or 2+, as depicted in Fig. 1. Convergence of a few vessels toward the tumour implant was denoted as 1+, and 2+ reflected an increased density and length of vessels converging toward the tumour implant.

Representative photographs were taken of tumours implanted on 7-, 9-, and 11-day-old embryos after injecting a large CAM vein with 0·5 ml of Pelikan ink (Gunther Wagner, Hanover, W. Germany) with a 30-g hypodermic needle (Cotran, Suter and Majno, 1967). The portion of the CAM containing the tumour was then excised, immersed in 10% formalin, and photographed while floating in a petri dish, using direct and indirect light.

*Large tumour implants*.—In a separate experiment, tumours 2, 3 and 4 mm in diameter were implanted on the CAM of 9-day-old chick embryos. The techniques previously described were used, except that the CAM was pierced with a No. 11 surgical blade in order to make a hole which would accommodate the implant.

*Histology*.—Chorioallantoic membranes containing tumour implants made between the 6th and the 11th day of embryonic life, were removed for fixation at one-day intervals from the 1st to the 10th day after implantation. The CAM was immersed for 2 h in modified Karnovsky's fixative (Karnovsky, 1965), (2·5% glutaraldehyde–2%

FIG. 1.—Grading of the CAM response to tumour implants was in three categories. A 0 response denoted no change in the CAM vasculature, convergence of a few vessels toward the tumour implant was a 1+ and a greater density and length of vessels was denoted 2+.

paraformaldehyde in 0·1M cacodylate buffer, pH 7·4) at 4°C. The membranes were washed overnight in cacodylate buffer containing 6% sucrose, post-fixed in buffered 1% osmium tetroxide, dehydrated and embedded in Epon. One micron-thick sections were stained with toluidine blue.

*Autoradiography.*—Implants of Walker 256 carcinosarcoma were made on the CAM of 14 5-day-old embryos and 6 7-day-old embryos. On each day after implantation, 2 eggs from each group received 50 $\mu$Ci of [$^3$H]thymidine (sp. act. 2·6 Ci/mmol; New England Nuclear, Boston, Mass.) in 0·5 ml of Medium 199, by injection into the allantoic sac. After 5 h of incubation, the CAM of each injected egg was excised, rinsed in Medium 199, fixed in Karnovsky's fixative and prepared for Epon embedding. Sections were prepared for autoradiography according to standard methods (Baserga and Malamud, 1969).

### RESULTS

Daily measurements were made on tumours implanted on CAMs (aged 5–13 days) in a total of 632 eggs. Of these, 339 were excluded, because either the embryo did not survive for 5 or more days (86 eggs), or the grafted tumour was displaced by the moving embryo and thus failed to take (106 eggs), or the graft was not viable tumour by gross examination and confirmed by representative histologic sections (147 eggs). Tumours implanted on 14- to 16-day-old CAMs failed to incorporate into the aging membrane and fell into the allantois, leaving a hole in the membrane.

### *Tumour growth*

All tumours implanted from Day 5 to Day 11 exhibited 2 phases of growth. During the first 72 h, tumours implanted at a size of approximately 1 mm remained near their implantation size (Fig. 2). Thereafter, they grew rapidly. Tumours implanted on Days 12 and 13 either remained at the original implantation size for the entire observation period, or shrank after 72 h (Fig. 2).

For the 5- to 11-day implants, the rate of tumour growth during the rapid growth phase and the final tumour diameter varied according to the day of implantation (Fig. 2). Tumours implanted on Days 5 and 6 grew most rapidly, and reached final diameters of up to 10 mm. Tumours implanted on Days 7, 8 and 9 grew more slowly, and reached final diameters of 3–5 mm, while those implanted on Days 10 and 11 grew slowest, reaching an average diameter of 1·5–2·5 mm.

### *Large implants*

Large tumours (2, 3 and 4 mm) implanted into the 9-day-old CAM, exhibited 2 distinct phases of growth (Fig. 3). During the first 72–96 h, all implants decreased in size. Tumours of 2 mm shrank to an average of 1·4 mm by 72 h, 3-mm implants shrank to an average of 1·9 mm by 72 h, while 4-mm tumours decreased their diameter to 2·4 mm by 96 h. After 72–96 h, all tumours underwent rapid growth, reaching average diameters of 5–7 mm.

### *Vascular response of the CAM*

Changes in the vasculature of the CAM in response to tumours (see Fig. 1) were not grossly observable until after the 10th day of incubation, regardless of the day of implantation. At this time, the membrane exhibited a positive response, *i.e.* a convergent set of vessels directed toward the implant. These readings are summarized in the Table.

### *Histology*

A detailed histological study was carried out and has been described in a separate report (Ausprunk, Knighton and Folkman, 1975). Only a summary is included here. All implants exhibited avascular and vascular growth phases. Tumours remained avascular without histologically discernible chick capillaries in the tumour mass for approximately 72 h. The duration of this avascular phase was the same for CAM implants made from Days 5 to 11.

FIG. 2.—Growth curves of Walker 256 rat carcinoma implanted on the CAM from Day 5–13. Arrows indicate the beginning of the rapid growth phase which occurs at approximately 72 h. Bars indicate standard deviations.

*Histologic avascular phase*

During the first 24 h, implants from Days 5 to 11 were incorporated into the CAM (Fig. 4). The implants lay contiguous to the mesodermal vessels of the CAM, but the vessels did not penetrate the tumour. Vessels that were originally part of the tumour graft disappeared after 24 h as seen by light microscopy of thick Epon sections. There were no endothelial cells visible within the tumours, and only a rare rat erythrocyte was observable on histological section. By 48 h, the central portion of implanted tumours became necrotic, leaving a peripheral shell of living cells which incorporated [$^3$H]-thymidine (Fig. 5).

In tumours implanted on Day 9 or later, CAM blood vessels near the implant contained increased numbers of small

FIG. 3.—Growth curves for 1-, 2-, 3- and 4-mm tumours implanted on the 9-day-old CAM. A rapid decrease in tumour diameter occurred during the first 96 h of incubation, followed by a rapid increase in size. Vascular penetration of the tumours occurs at 72 h post-implantation.

granular and agranular monocytic cells. One to 2 days after grafting, these cells together with chick mesodermal cells were concentrated at the tumour margins, and appeared to encapsulate the implants.

*Histologic vascular phase*

After 72 h, blood vessels from the CAM penetrated most of the tumours examined (Fig. 6). Healthy tumour cells which incorporated [$^3$H]thymidine were present in the vascular areas of the tumour (Fig. 7). By 96 h, in implants made on 5- to 8-day CAMs, viable tumour cells and many small blood vessels were seen throughout the tumour. Seven or 8 days post-implantation, these tumours had doubled or tripled in size, although blood vessels and tumour cells at the centre of the growing implant began to degenerate. Implants made on Day 9 or later were also vascularized in 3 days. At this time, however, implants were infiltrated with chick mesodermal cells and large, debris-laden phagocytes. Thereafter tumours were gradually replaced by chick mesodermal tissue.

### DISCUSSION

These experiments provide further evidence for the concept that tumour angiogenesis is a control mechanism in tumour growth. (1) Tumour growth could be divided into an initial avascular phase followed by a vascular phase. (2) Neovascularization occurred at 72 h regardless of the day of implantation. (3) Rapid growth occurred only after neovascularization, and was modulated by the age of the embryo at the time of tumour implantation.

Although previous studies (Gimbrone *et al.*, 1972; Folkman, Cole and Zimmerman, 1972; Folkman and Hochberg, 1973) have shown that tumours maintained in the avascular state will form dormant spheroids or ellipsoids, and not enlarge beyond approximately 1 mm, each of these experiments was subject to the criticism that the tumour was either suspended in a moving fluid far from host vessels, or apposed to vessels incapable of proliferation. We now show that tumour implants completely surrounded by healthy vessels still do not grow until penetrated by new vessels. Furthermore, this is the first documentation of the time of onset of the vascular phase and its early events in the chick embryo. Even though this period is brief, *i.e.* 72 h, the behaviour of the tumours, histologically and grossly, are similar to avascular spheroidal tumour growth in three previous reports. Thus, in the avascular phase, tumours appeared spheroidal. They did not spread out as flat plates along the CAM. The size of the tumour at the end of the avascular phase was relatively constant (about 1 mm). These tumours consisted of a population of

TABLE.—*Grading of the Response of CAM Blood Vessels to Tumour Implants Made from Day 5 to 13*

| Day of implantation | Membrane response | Day of reading 7 | 8 | 9 | 10 | 11 | 12 | 13 | 14 | 15 | 16 | 17 |
|---|---|---|---|---|---|---|---|---|---|---|---|---|
| | | Number of eggs exhibiting indicated response | | | | | | | | | | |
| 5 | 0 | 56 | 62 | 57 | 44 | 24 | 16 | 2 | 1 | 2 | 6 | 4 |
| | +1 | 0 | 0 | 0 | 6 | 17 | 21 | 18 | 13 | 8 | 2 | 0 |
| | +2 | 0 | 0 | 0 | 0 | 0 | 0 | 0 | 2 | 2 | 0 | 0 |
| 6 | 0 | 30 | 30 | 29 | 17 | 5 | 2 | 1 | 1 | 1 | 1 | 2 |
| | +1 | 0 | 0 | 0 | 10 | 20 | 21 | 17 | 9 | 4 | 2 | 2 |
| | +2 | 0 | 0 | 0 | 0 | 0 | 1 | 3 | 11 | 12 | 10 | 7 |
| 7 | 0 | | 30 | 28 | 23 | 9 | 0 | 1 | 1 | 1 | 0 | 0 |
| | +1 | | 0 | 0 | 2 | 10 | 9 | 6 | 4 | 3 | 4 | 3 |
| | +2 | | 0 | 0 | 0 | 1 | 7 | 7 | 8 | 7 | 3 | 2 |
| 8 | 0 | | | 32 | 28 | 22 | 6 | 5 | 2 | 0 | 0 | 0 |
| | +1 | | | 0 | 0 | 4 | 17 | 14 | 11 | 5 | 4 | 3 |
| | +2 | | | 0 | 0 | 2 | 3 | 4 | 7 | 8 | 6 | 6 |
| 9 | 0 | | | | 30 | 24 | 11 | 2 | 1 | 1 | 1 | 0 |
| | +1 | | | | 0 | 1 | 9 | 12 | 5 | 4 | 4 | 4 |
| | +2 | | | | 0 | 0 | 0 | 1 | 8 | 6 | 5 | 3 |
| 10 | 0 | | | | | 29 | 26 | 5 | 9 | 5 | 1 | 1 |
| | +1 | | | | | 0 | 0 | 20 | 11 | 10 | 10 | 7 |
| | +2 | | | | | 0 | 0 | 1 | 4 | 8 | 8 | 10 |
| 11 | 0 | | | | | | 31 | 17 | 11 | 3 | 2 | 0 |
| | +1 | | | | | | 0 | 13 | 17 | 19 | 14 | 12 |
| | +2 | | | | | | 0 | 0 | 0 | 4 | 8 | 9 |
| 12 | 0 | | | | | | | 10 | 10 | 8 | 6 | 3 |
| | +1 | | | | | | | 6 | 6 | 5 | 5 | 3 |
| | +2 | | | | | | | 0 | 0 | 2 | 1 | 3 |
| 13 | 0 | | | | | | | | 10 | 9 | 7 | 2 |
| | +1 | | | | | | | | 0 | 1 | 2 | 5 |
| | +2 | | | | | | | | 0 | 0 | 0 | 0 |

centrally necrotic cells, together with proliferating cells at the periphery. Tumour implants 2 mm and larger shrank rapidly during the avascular phase, but were 1·4–2·5 mm at 72 h when vascular penetration occurred. Thus, the behaviour of implants during the avascular period *in vivo* resembled the growth of tumours *in vitro*, where transport of nutrients and wastes occurs only by diffusion (Folkman, Hochberg and Knighton, 1974).

During the 72-h avascular phase, rat blood vessels within the tumour transplants disintegrated by approximately 24 h (Ausprunk and Folkman, 1976). A number of events probably occur which lead to the penetration of the tumour by new host vessels. These include the envelopment of the tumour by the host membrane, production of an angiogenic factor (TAF) by the tumour, the endothelial cell response to TAF and the formation of new capillaries which travel toward the centre of the tumour. The time of onset of the vascular phase seemed to be independent of the immunological status of the embryo. Thymus cells are present by Day 11, and cell-mediated immunity has been demonstrated by Day 13–14 (Solomon, 1971). However, the onset of angiogenesis occurred after 72 h regardless of the day of implantation.

The vascular phase was marked by three significant events. The first was the rapid tumour growth which began between 72 and 96 h (Fig. 3). The second was the disappearance of the central necrosis which had been present in the previous avascular phase. As tumours approached 3–4 mm, central necrosis reappeared, probably because the deeper vessels were compressed by the expanding tumour mass (Goldacre and Sylven, 1959; Young, Lumsden and Stalker, 1950). The third

FIG. 4.—Chick CAM 24 h after implantation of Walker 256 tumour on Day 6. Endodermal cells (En) of the CAM have already covered the lower portion of the tumour, while the CAM ectoderm (Ec) is still discontinuous. No blood vessels are present within the tumour, but mesodermal vessels (arrowed) of the CAM are located at its periphery. × 85.

FIG. 5.—Autoradiograph of an avascular tumour 2 days after implantation on a 7-day CAM. Tumour cells at the periphery of the implant have incorporated [$^3$H] thymidine while those at its centre have not. × 165.

FIG. 6.—Vascularized tumour 3 days after implantation on a 7-day CAM. Most of the tumour is penetrated by blood vessels containing chick erythrocytes. × 165.

FIG. 7.—Autoradiograph of a tumour implanted 3 days earlier on a 7-day CAM. Tumour cells which have incorporated [$^3$H]thymidine are located in the portion of the tumour which has been penetrated by chick blood vessels (arrows). Cells farthest from the capillaries appear necrotic, and have not incorporated [$^3$H] thymidine. × 625.

was a gradual decrease in the slopes of the tumour growth curves as the age of the implanted embryo increased.

The gradual decrease in the rate of tumour growth as a function of the day of implantation may be related to the immunological state of the embryo. The presence of monocytic cells at the periphery of tumours implanted after Day 9 supports this possibility. The onset of immunity could modify the rate of tumour growth, while at the same time the beginning of rapid growth is determined by the penetration of new vessels. These two phenomena, angiogenesis and immunity, may operate independently in their effects upon tumour growth.

An alternative explanation is that as the CAM ages or matures, the growth of the tumour might slow down, due to a decrease in the amount of blood being supplied to the CAM. The CAM does begin to degenerate between Days 17 and 19 (Zwilling, 1959; Hamburger, 1960), but this would seem to occur too late to account for the steadily decreasing rate of tumour growth observed for implants from Day 5 to 13.

A separate study of the normal mitotic activity of the CAM endothelium, made in our laboratory (Ausprunk, Knighton and Folkman, 1974), could explain why a membrane response to implanted tumours is not grossly observable until after the 10th or 11th day of incubation. The CAM endothelium exhibits an intrinsically high mitotic rate (thymidine labelling index of 23% for 5-h thymidine exposure) until Day 10. At the 11th day of incubation this labelling index falls to 2% and remains low throughout the remaining incubation period. Thus any angiogenic stimulus might be masked by the normally high background activity of growing vessels, up to the 11th day. After this, when vessel growth slows down, the altered pattern of the CAM vessels in response to an angiogenic factor might be more easily detected. This capacity of the CAM to display a vascular response after 11 days permits us to use it as an assay for TAF fractions.

Finally, although there are many reports on the use of the chick CAM for tumour growth, this is the first study in which the parameters of tumour growth have been quantitated over such a wide range of implantation times, *i.e.* throughout almost the entire incubation period of the chick embryo. This will provide a standard of comparison for future studies of the effects of angiogenesis, immunity, tolerance, radiation, and implant size, on tumour growth. These data should also provide a standard for future studies of possible inhibitors of tumour angiogenesis.

We thank Dr Robert Auerbach and Dr Ramzi S. Cotran for helpful advice, Mrs Gwladys Caspar for technical assistance, Ms Christine Keller for histological assistance, Mr Janis Cirulis for medical art, and Mrs Pauline Breen for typing the manuscript.

This work was supported by a grant from the National Cancer Institute (No. 1RO1-CA 14019-01), a grant from the American Cancer Society (No. DT-2A), and gifts from Alza Corporation, Merck Co., and Mr Morton Bank.

### REFERENCES

AUSPRUNK, D. H. & FOLKMAN, J. (1976) Vascular Injury in Transplanted Tissues: Fine Structural Changes in Tumor, Adult and Embryonic Blood Vessels. *Virchows Arch. B. Cell Path.*, **21**, 31.

AUSPRUNK, D. H., KNIGHTON, D. R. & FOLKMAN, J. (1974) Differentiation of Vascular Endothelium in the Chick Chorioallantois: A Structural and Autoradiographic Study. *Dev. Biol.*, **38**, 237.

AUSPRUNK, D. H., KNIGHTON, D. R. & FOLKMAN, J. (1975) Vascularization of Normal and Neoplastic Tissues Grafted to the Chick Chorioallantois. *Am. J. Path.*, **79**, 597.

BASERGA, R. & MALAMUD, D. (1969) Microscopic Autoradiography. In: *Autoradiography: Techniques and Application.* New York: Harper and Row.

COTRAN, R. S., SUTER, E. & MAJNO, G. (1967) On the Use of Colloidal Carbon as a Tracer for Vascular Injury. *Vasc. Dis.*, **4**, 107.

FOLKMAN, J. (1974) Tumor Angiogenesis. In: *Advances in Cancer Research.* Eds. G. Klein and S. Weinhouse. New York: Academic Press.

FOLKMAN, J., COLE, P. & ZIMMERMAN, S. (1972) Tumor Behavior in Isolated Perfused Organs: *In vitro* Growth and Metastases of Biopsy Material in Rabbit Thyroid and Canine Intestinal Segment. *Ann. Surg.*, **175**, 408.

FOLKMAN, J. & HOCHBERG, M. (1973) Self-Regulation of Growth in Three Dimensions. *J. exp. Med.*, **138**, 745.

FOLKMAN, J., HOCHBERG, M. & KNIGHTON, D. (1974) Self-Regulation of Growth in Three Dimensions: Role of Surface Area Limitation. In: *Control of Proliferation in Animal Cells.* Eds. B. Clarkson and R. Baserga. New York: Cold Spring Harbor Laboratory.

GIMBRONE, M. A., LEAPMAN, S. B., COTRAN, R. S. & FOLKMAN, J. (1972) Dormancy *In vivo* by Prevention of Neovascularization. *J. exp. Med.*, **136**, 261.

GOLDACRE, R. J. & SYLVEN, B. (1959) A Rapid Method for Studying Tumor Blood Supply Using Systemic Dyes. *Nature, Lond.*, **184**, 63.

HAMBURGER, V. (1960) *A Manual of Experimental Embryology.* Chicago: University of Chicago Press.

KARNOVSKY, M. J. (1965) A Formaldehyde-Glutaraldehyde Fixative of High Osmolarity for Use in Electron Microscopy. *J. Cell Biol.*, **27**, 137A.

SOLOMON, J. B. (1971) Lymphocytopoeisis and Ontogeny of Defined Immunity in Birds. In *Fetal and Neonatal Immunology.* New York. Frontiers of Biology, Monograph 20.

YOUNG, A. S., LUMSDEN, C. E. & STALKER, A. L. (1950) The Significance of Tissue Pressure of Normal Testicular and of Neoplastic (Brown-Pearce) Carcinoma Tissue in the Rabbit. *J. Path.*, **62**, 313.

ZWILLING, E. (1959) A Modified Chorioallantoic Procedure. *Transplant. Bull.*, **6**, 115.